# Design and Validation of an Open–Close Device for Integrated Environmental DNA Sampling Detects A Depth Gradient in Indian Ocean Deep‐Sea Fish Assemblages

**DOI:** 10.1002/ece3.70902

**Published:** 2025-01-28

**Authors:** Cindy Bessey, Andrew Martini, Alasdair Currie, Will Ponsonby, Aaron Tyndall, Ryan Crossing, Vinícius Werneck Salazar, Kathryn L. Dawkins, John J. Pogonoski, Glenn Moore, Nick Mortimer, John K. Keesing

**Affiliations:** ^1^ Commonwealth Scienctific and Industrial Research Organisation Indian Ocean Marine Research Centre Crawley Western Australia Australia; ^2^ Commonwealth Scientific and Industrial Research Organisation National Collection and Marine Infrastructure Hobart Tasmania Australia; ^3^ Commonwealth Scientific and Industrial Research Organisation National Collection and Marine Infrastructure Crawley Western Australia Australia; ^4^ Melbourne Integrative Genomics University of Melbourne Parkville Victoria Australia; ^5^ eDNA Frontiers, School of Molecular and Life Sciences Curtin University Bentley Western Australia Australia; ^6^ Commonwealth Scientific and Industrial Research Organisation Australian National Fish Collection Hobart Tasmania Australia; ^7^ Fish Section, Department of Aquatic Zoology Western Australian Museum Welshpool Western Australia Australia; ^8^ School of Biological Sciences University of Western Australia Nedlands Western Australia Australia; ^9^ School of Molecular and Life Sciences Curtin University Bentley Western Australia Australia

**Keywords:** eDNA, fabrication, fishes, metabarcoding, monitoring, water sampling

## Abstract

Advances in methods for collecting environmental DNA (eDNA) are revolutionizing biomonitoring capabilities. The goal of this study was to leverage existing survey technology to design and test an eDNA sampler that captures an integrated eDNA sample over the length of a deep‐water transect. We manufactured a 300 × 100 × 100 mm mountable, open‐ended box made of high‐density polyethylene that could be attached to the frame of a preexisting deep tow camera system. The box (OCD; open–close device) was equipped with an actuator that attached to hinged doors at both ends, enabling it to be opened and closed remotely at depths up to 6000 m through preexisting communications, thereby exposing the internal chamber to the surrounding water upon activation. A sterile active carbon sponge was inserted into the internal chamber for eDNA capture during each deployment. The OCD sampler was field tested during a voyage to the Gascoyne Marine Park region off northwest Australia. We compared three different methods for processing the captured eDNA from the sampler: filtering OCD water, extracting eDNA from sponge pieces, and filtering sponge rinse water. Using fish as our example organism, we also compared the identities of fishes from eDNA detections with bottom trawl survey data collected during the same survey, and the known regional species pool, to confirm the eDNA identifications were plausible. A large number of fishes (193 taxa, from 87 families) were detected, and the majority were found within their expected depth ranges (> 75%), and in the trawl catches (60%). We discuss design and manufacturing lessons, ideas for increased eDNA capture efficiency for improved methodologies in sample processing, and how to establish appropriate field controls. We also discuss how this technology could advance our scientific understanding in ocean studies in terms of ecological metrics provided and the trade‐offs compared to other sampling tools.

## Graphical abstract

The goal of this study was to leverage existing survey technology to design and test an eDNA sampler that captures an integrated eDNA sample over the length of a deep‐water transect. We provide a biological interpretation of the resulting data, demonstrating the usefulness of such technology to understand the ocean environments, using fish species as our example organisms. We discuss design and manufacturing lessons, ideas for increased eDNA capture efficiency for improved methodologies in sample processing, how to establish appropriate field controls, and how this technology could advance our scientific understanding in ocean studies.

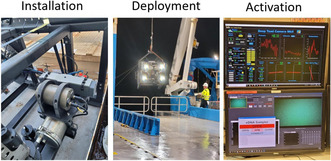

## 1 Introduction

Biomonitoring capabilities are being revolutionized through environmental DNA (eDNA) advances that are rapidly becoming more diverse (Takahashi et al. 2023). The collection, extraction, and identification of DNA in air, land, and waterbodies have led to efficient and sensitive methods to survey species biodiversity with increasing accuracy (Taberlet et al. 2018; Thomsen and Willerslev 2015). In aquatic environments, active filtration of water samples collected by people is the most widely used technique (Takahashi et al. 2023; Tsuji et al. 2019; Zinger et al. 2019), although new methodological developments are quickly emerging (McQuillan and Robidart 2017), such as high‐volume tow‐nets (Schabacker et al. 2020), passive eDNA collection devices that use natural water movement or vessel movement (Jeunen et al. 2024; Bessey et al. 2021; Kirtane, Atkinson, and Sassoubre 2020), automated in situ samplers with inbuilt analytical platforms (Scholin et al. 2017) and permanent networks of seabed and water‐column‐cabled (fixed), and docked mobile platforms and pumping systems (Canals et al. 2021; Aguzzi et al. 2019). These technological advancements (e.g., autonomous sensors) offer cost savings, and high‐frequency sampling for remote and hard‐to‐access environments and have been used in both marine and freshwater applications of biodiversity, detection of human pathogens, and invasive species surveillance (Sepulveda, Birch, et al. 2020; Sepulveda et al. 2019; Sepulveda et al. 2020; Yamahara et al. 2019, 2015).

Deep seas are among the least explored and most pristine ecosystems remaining on Earth (Martin et al. 2020) and are an example of challenging environments where advances in eDNA methodologies are proving both useful and necessary in improving our biodiversity knowledge, as interest in acquiring deep sea resources increases (Canals et al. 2021; Aguzzi et al. 2019). Typically, a rosette of large bottles (often 5, 8, 10, or 12 L), known as Niskin bottles, are lowered to the depth at a particular location where they are closed through remote activation, and the collected water is retrieved for eDNA studies (Canals et al. 2021; Visser et al. 2021; Laroche et al. 2020; McClenaghan et al. 2020). In the Bay of Biscay, Northeast Atlantic Ocean, eDNA metabarcoding analysis on Niskin samples from up to 2000 m in depth revealed 25 species of deep‐sea fish with deep‐sea species richness increasing with depth (Canals et al. 2021). Another approach is to use water samples from deep‐sea pumping facilities or from cabled observatories, which are seabed oceanographic platforms equipped with biogeochemical and oceanographic sensors and internet‐operated vehicles that can crawl or move around on the seafloor before returning to their docking stations (Yoshida et al. 2023; Stefanni et al. 2022; Mirimin et al. 2021). For example, researchers on the Pacific coast of Japan filtered large quantities of water from a pumping facility (up to 800 m depth) to determine the optimal filtration volume and replication suitable for continuous fish biodiversity monitoring at depth (Yoshida et al. 2023). Their study showed the importance of large‐volume filtration (20 L) to increase deep‐sea species detection, where they were able to detect 92 fish species, with a further 24 identified to family level. The need for large‐volume sampling has likewise been demonstrated in shallow water eDNA studies, due to the heterogeneous and patchy nature of eDNA (Bessey et al. 2020), but this is particularly pronounced in the deep‐sea where biomass is low, therefore, resulting in lower eDNA concentrations (McClenaghan et al. 2020).

The goal of our current study was to leverage existing deep‐water survey technology to design and test a deep‐water eDNA sampler that primarily uses passive collection to potentially capture increased DNA concentrations. We designed an eDNA sampler that affixes to a deep‐sea towed underwater camera; an imaging system that is considered critical technology for national research vessels to obtain information about the marine environment (Sherlock et al. 2016). This platform is especially useful in marine sanctuaries, where extractive methods may be constrained. The camera system can be towed along transects at slow speed (< 2 knots) 2 m above the seafloor at depths of up to 4000 m and is equipped with oceanographic sensors for conductivity, temperature, and depth. When initially used in combination with extractive methods that enable specimen retention, and the expertise of experienced taxonomists, the combination provides a complementary ground‐truthing opportunity that allows for accurate identification of organisms both morphologically and genetically and will enable image analysis specialists to determine the level of classification obtainable from the imagery. This study outlines the design and execution of a passive eDNA sampler attachment to a deep‐sea towed camera system and tests its feasibility to characterize species assemblages along the length of a transect. Using fish as our example organism, and bottom‐trawl survey data collected concurrently in the study area for comparison, we outline the capabilities and limitations of a new deep‐sea passive eDNA sampler affixed to a camera system.

## 2 Materials and Methods

### 2.1 Design of an eDNA Sampler

**FIGURE 1:**
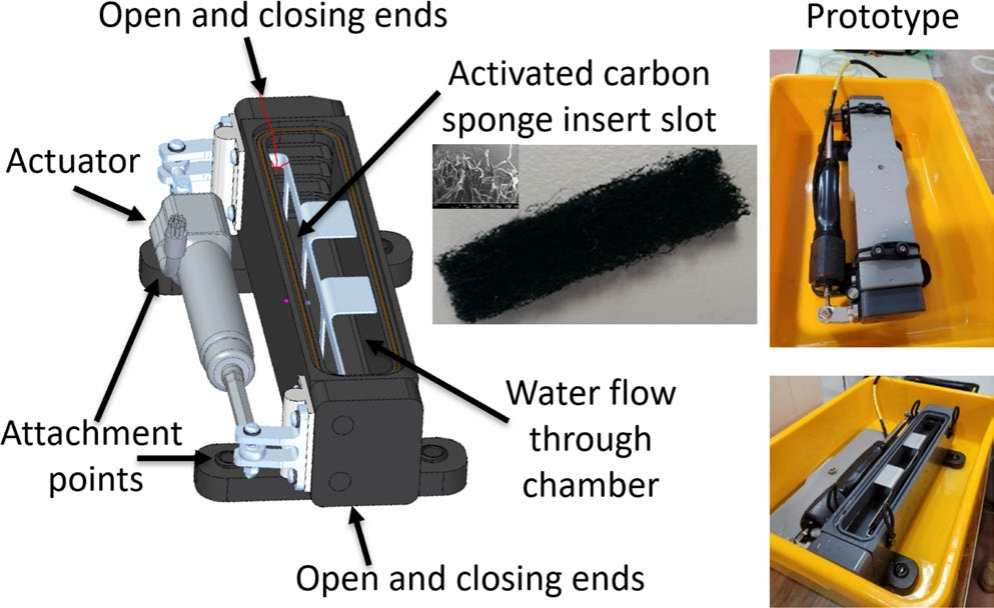
Schematic of the OCD (open–close device) eDNA sampler featuring key components, including the sponge capture material and photographs of the manufactured prototype with lid on and lid off.

**FIGURE 2:**
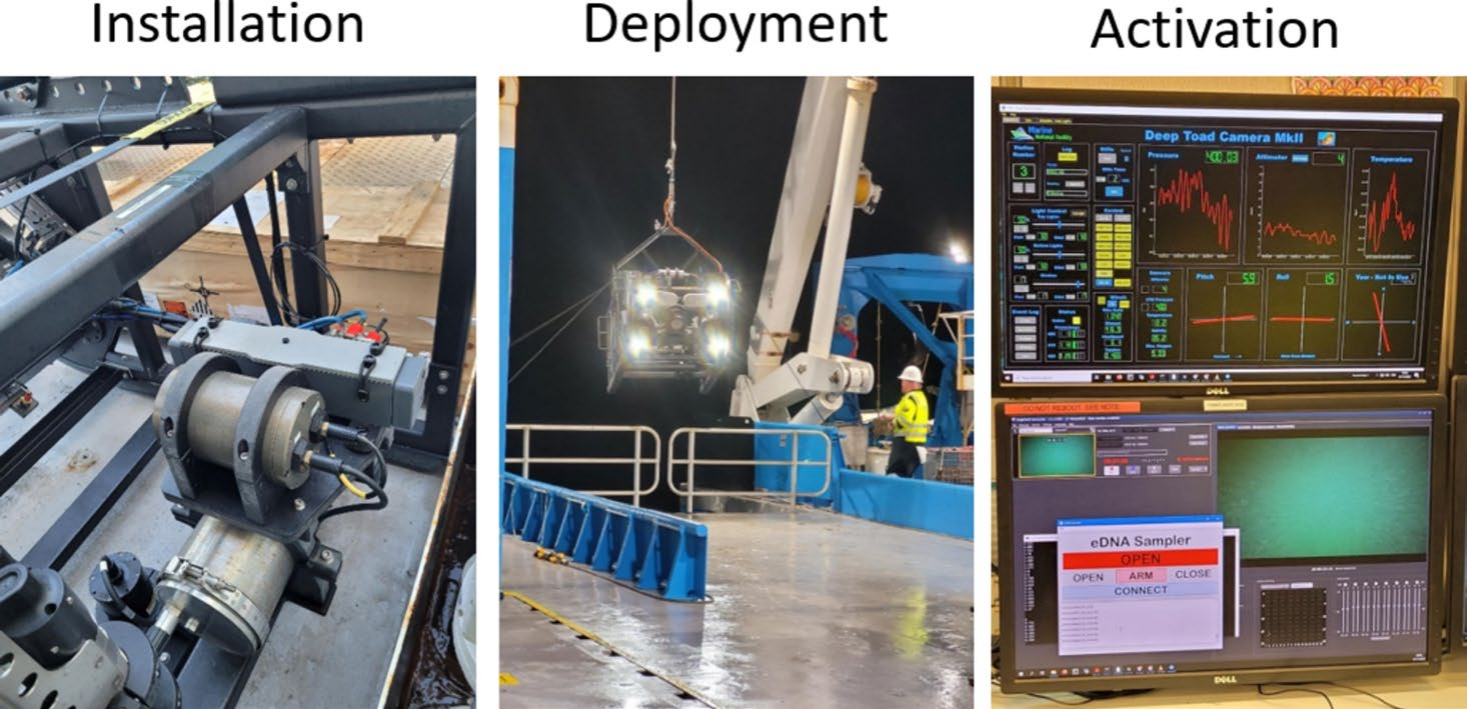
Photographs showing the installation position of the OCD on the deep‐tow camera system frame, deployment of the combined OCD and camera system, and activation screen of the software that enables the OCD to be opened and closed remotely.

The OCD (open–close device) sampler was designed to capture an eDNA sample over the length of a transect at depth. Unlike a Niskin bottle, which is typically used to collect water at one location and depth, we aimed to maximize DNA collection over the length of a transect and at the same time as imagery of the seabed and associated fauna was being collected. We manufactured a 300 × 100 × 100 mm mountable, open‐ended box made of high‐density polyethylene that could be attached to the frame of a preexisting deep tow camera system (Figures 1 and 2; Sherlock et al. 2016). The box was equipped with an actuator (Ultramotion; www.ultramotion.com; 6000 m rating) that attached to hinged doors at both ends, enabling it to be opened and closed remotely at depth though preexisting communication (6000+ m electro‐optical wire), a programmable motor controller, and software for the deep tow camera system, thereby exposing the internal chamber to the surrounding water upon activation. Each end was fitted with a rubber pad and nitrile O‐rings to facilitate sealing when closed. The internal chamber of the box was left unmodified to accommodate a variety of collection materials, with a holding capacity of approximately 1.2 L. The lid contained a pressure relief valve to prevent implosion should air bubbles occupy the internal chamber during deployment. The lid contained an elastic restraining band to secure it in position against a sealing O‐ring. The OCD sampler was fitted with four attachment points for mounting on the deep tow camera frame and the total weight of the OCD was approximately 7 kg empty.

### 2.2 Installation Onto the Deep Tow Camera Frame

The OCD sampler was installed on the back of the deep tow camera frame by Proflow quick‐release mounts with the actuator power cable attached to the closed motor controller (Figure 2). The vessel crew deployed the deep tow camera system using the vessel A‐frame (Triplex 20 t, height 9.4 m, width 5.3 m, 170° swing range) and the general purpose winch. The deep tow camera system was lowered and piloted at approximately 2 m above the seafloor, which could be monitored in the operations rooms through the live camera video feed and altimeter readings. The OCD actuator was then activated from the ship in the operations room through an application that worked in conjunction with the preexisting software system, causing the ends of the OCD device to open at depth allowing the flowthrough of water over the duration of the transect. The deep tow camera system, with OCD, was towed at < 2 knots along an approximate 2 km transect (~30 min per transect). At the end of the transect, but before ascent of the camera system, the actuator was again activated to close the doors of the OCD sampler capturing any water that remained within the box compartment. Upon completion of the transect, the deep tow camera system was retrieved and returned to the deck of the vessel where the OCD sampler could be retrieved for sample processing. In summary, the OCD remained closed on the descent to and ascent from depth and was only opened once at depth for the length of the transect.

### 2.3 Field Program and Sample Collection

**FIGURE 3:**
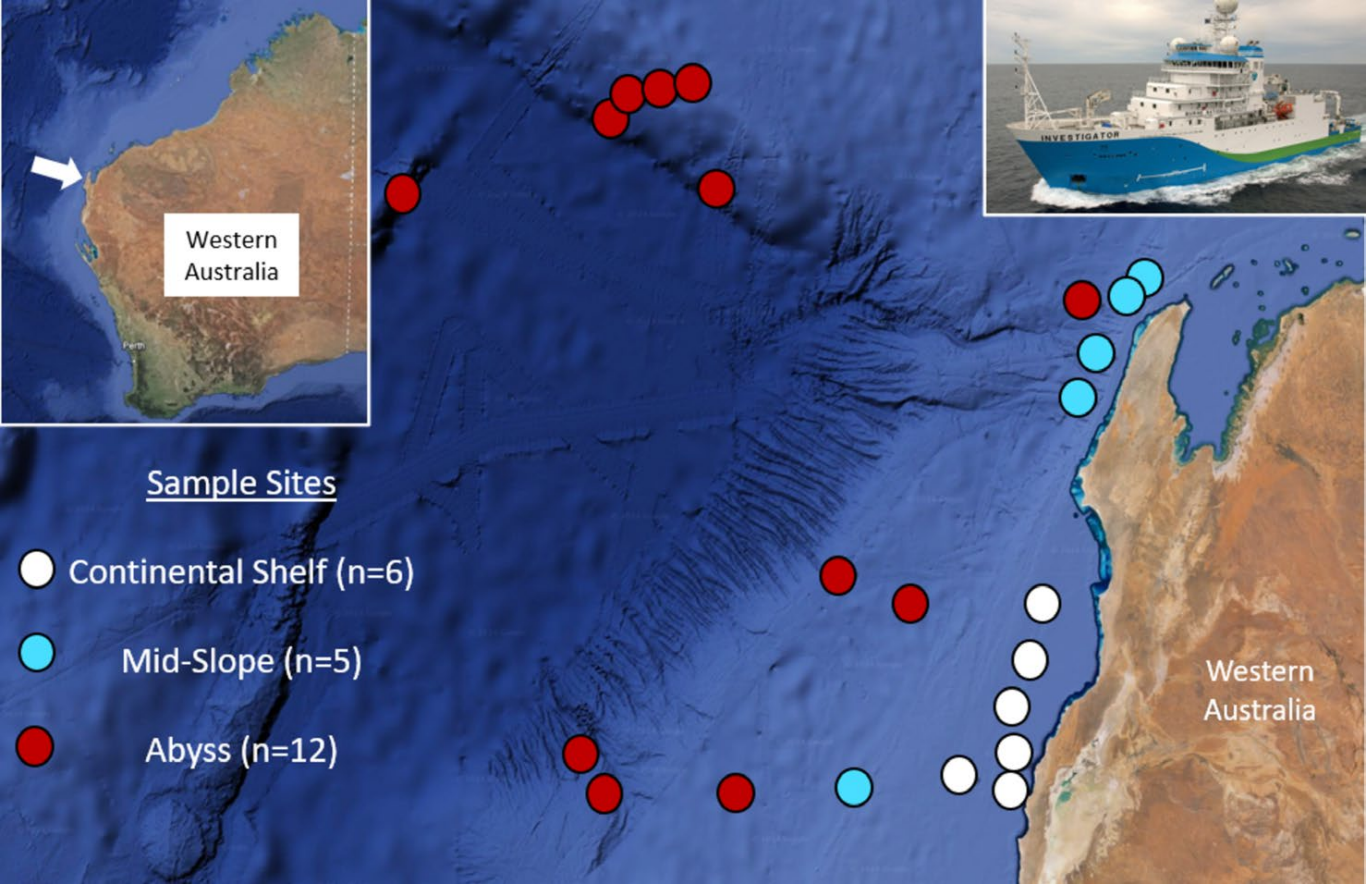
Google Earth map of the sample site locations off the north‐west coast of Western Australia by depth category (white circle = continental shelf, blue circle = mid‐slope, and red circle = abyss), with an inserted photograph of the RV Investigator.

The OCD sampler was field tested aboard the RV Investigator, a 93.9 m vessel managed and operated by the Commonwealth Scientific and Industrial Research Organisation (CSIRO), through Australia's Marine National Facility (Figure 3). The work was conducted during a voyage to the Gascoyne Marine Park region in northwest of Western Australia, led by Chief Scientist John Keesing in December 2022. Samples were successfully obtained from deep‐tow camera OCD single deployments at 23 sites (*n* = 23), where each site represented one discrete depth, but sites ranged across several water depths categorized as continental shelf (0–200 m; *n* = 6), shelf break to mid‐slope (200–1000 m; *n* = 5), and mid‐slope to abyss (1000–4000 m; *n* = 12). All OCD deployments are detailed in Appendix S1. The deep‐tow camera OCD eDNA transects were paired to trawl sampling at the same depth and sites conducted as close as possible to the line and length of the OCD transects. Trawling was always conducted after the OCD transects so as not to stir up the bottom sediments and potentially affect the results of the eDNA sampling. Trawling operations were carried out using both a beam trawl (4 m wide, 0.5 m high, 10 mm cod end mesh, tow length ~ 2000 m) and a modified scampi style demersal trawl net (32 m wide, 3 m high, 50 mm cod end mesh, tow length ~ 2000 m). The latter could only be used effectively at sites with depths down to 1500 m, while the beam trawl was used at all sites down to > 5000 m. Expert fish taxonomists identified specimens as best as possible onboard and upgraded identification in the laboratory following the voyage where required. The resulting data from samples obtained by the OCD eDNA sampler were compared to the fish species observed from the trawl catches.

### 2.4 On‐Board OCD Sampler Preparation

We provide a detailed standard operating procedure in Appendix S2 for eDNA collection using the OCD, which is also summarized here. To prepare the OCD for deployment, it was placed open in 10% bleach water for 30 min to sterilize the entire unit, including the lid, after which time it was rinsed with deionized water and placed in a sterile carry tray for easy transport (Figure 1). A sterile active carbon sponge was inserted into the OCD for eDNA capture during each deployment. The OCD was then filled with deionized water allowing for slight overflow (just over 1 L) ensuring no air bubbles remained and the lid was then secured. The prepared OCD was then transported to the sea‐going instrumentation team for attachment to the deep tow camera frame.

The sponge material (Aqua One, Kong's Pty Limited in Australia) was chosen because it is a tightly woven filter pad with 100% active carbon embedded into the fibers that is known to rapidly capture DNA (Figure 1; Bessey et al. 2022). Each sponge insert was approximately 30 × 6 × 0.5 cm. Sponge inserts were sterilized by soaking in 10% bleach for at least 30 min, allowed to dry in a sterilized cabinet, placed in an ultraviolet sterilizing cabinet for a minimum of 30 min, and then stored in a sterile zip‐lock bag for transport and storage aboard the vessel.

Upon retrieval, the OCD was stored in a 4°C fridge until the sample could be processed, which occurred within 4 h. The lid of the OCD was removed, the sponge insert was retrieved with sterile tweezers, and all excess water from the sponge was allowed to drain back into the OCD internal compartment. The sponge was then allowed to dry briefly (< 10 min to remove any remaining water ensuring eDNA was from sponge rather than water) in a sterile cabinet before being put into a labeled, sterile zip‐lock bag and stored in the −80°C freezer for the duration of the voyage. The remaining water in the OCD was then poured into a sterile 1 L container and filtered over a cellulose ester membrane (47 mm diameter, 0.45 μm pore size) using a peristaltic Sentino (Cytiva; formerly Pall Laboratories) Microbiology Pump. The resulting filter membrane was then rolled into a 2 mL Eppendorf tube and stored in the −80°C freezer for further processing at the onshore molecular laboratory.

**FIGURE 4:**
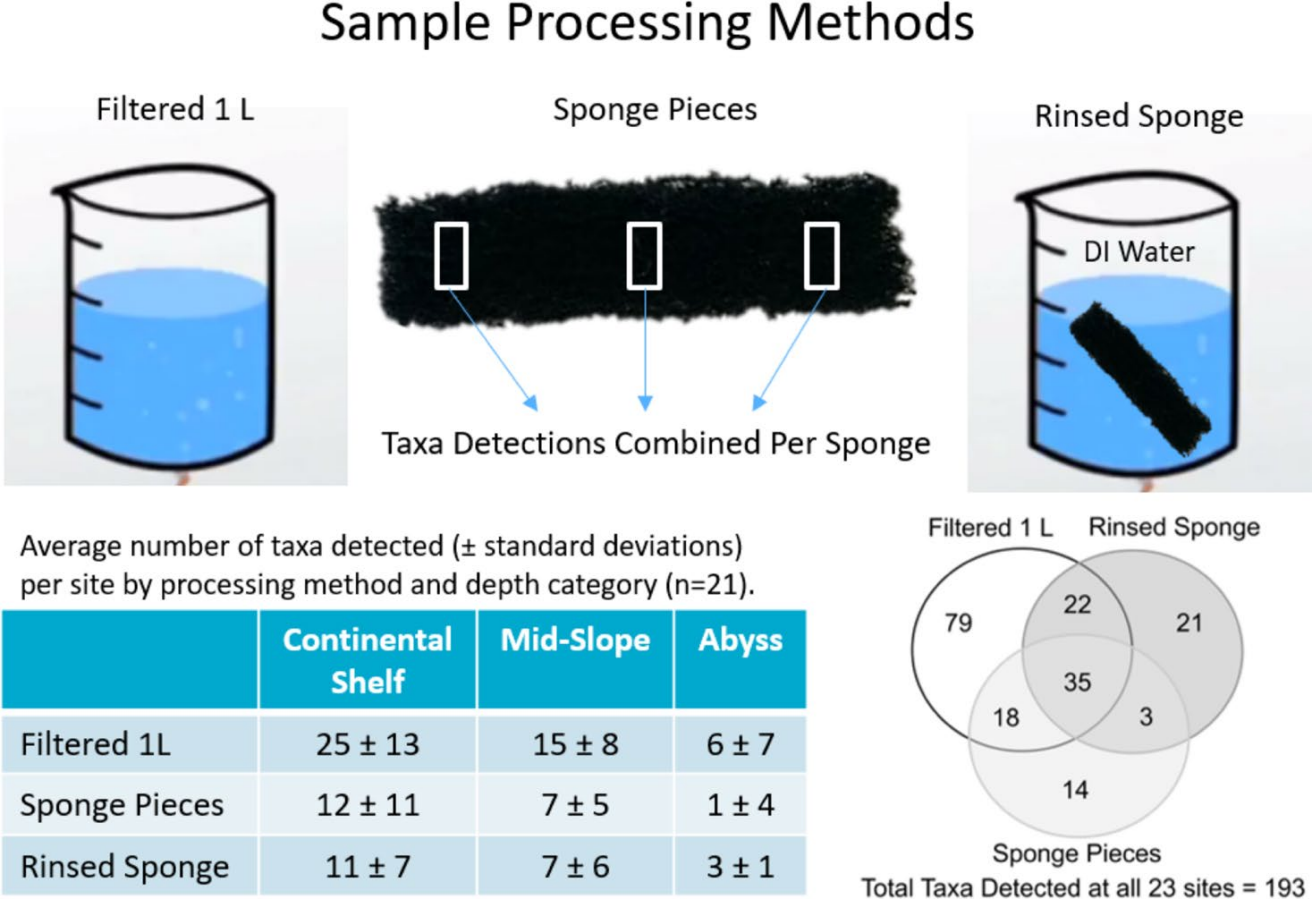
A graphical depiction of the three sample processing methods (filtered 1 L OCD water, sponge pieces as depicted by white rectangles, and rinsed sponge) used to compare fish species detection and a summary of the average number of taxa detected per site by processing method and depth category. Venn diagram depicts the total taxa detected for filtered 1 L samples compared to the taxa detected by the sponge pieces and rinsed sponge combined.

### 2.5 Contamination Control

The preparation of the OCD, water filtration, and storage of samples was done exclusively by two handlers within the vessel's clean dry laboratory who always remained gloved. All equipment was regularly sterilized in a 10% bleach solution for at least 15 min and rinsed in deionized water. All benchtops and cabinets were frequently wiped down with a bleach cloth.

### 2.6 Laboratory Processing of Samples

All sponge inserts were thawed and three small pieces (with each piece ~40 × 5 × 5 mm) of sponge were cut out of each insert (one from each end and one from the center) with sterile scissors and tweezers and placed in individually labeled 2 mL Eppendorf tubes (Figure 4). The sponge insert was then placed back into its zip‐lock bag with 1 L of deionized water where it was agitated by shaking the bag by hand for approximately 1 min to mechanically dislodge any DNA from the sponge into solution. The solution was then filtered over a cellulose ester membrane in the same fashion as described for the filtered OCD sample, with the resulting membrane rolled into a 2 mL Eppendorf tube.

Total nucleic acid was extracted directly from all samples in the Eppendorf tubes using the standard DNeasy Blood and Tissue Kit (Qiagen; Venlo, Netherlands) protocol, with an additional 40 μL of Proteinase K used during a 3‐h digestion period at 56°C on rotation (300 rpm). DNA was eluted into 200 μL AE buffer. All extractions took place in a dedicated DNA extraction laboratory (CSIRO Genomic Laboratory, Crawley, WA), where benches and equipment were routinely bleached and cleaned.

The purpose of comparing the 1 L water sample remaining in the OCD to that of the combined sponge pieces and the sponge rinse water (Figure 4) was to gain insight into the most effective method for processing the eDNA captured within the sampler. Ideally, the combination of these samples would be compared to a more conventional sampling method, such as processing water from a Niskin bottle sampled at the same location and depth; however, this was not an option on the current voyage. We hypothesized that eDNA would be concentrated on the sponge pieces.

### 2.7 DNA Amplification for Fish Detection

We followed the same procedures used by Bessey et al. (2021) and provide those details again here for reference. One‐step quantitative polymerase chain reactions (qPCR) were performed in duplicate for each sample using 2 μL of extracted DNA and a mitochondrial DNA 16S rDNA universal primer set targeting fish taxa (16SF/D 5′ GACCCTATGGAGCTTTAGAC 3′ and 16S2R‐degenerate 5′ CGCTGTTATCCCTADRGTAACT 3'with a 178–228 bp amplicon size; Berry et al. 2017; Deagle et al. 2007), with the addition of fusion tag primers unique to each sample that included Illumina P5 and P7 adaptors. Quantitative PCR was performed in a dedicated PCR laboratory. Quantitative PCR reagents were combined in a dedicated clean room and included 5 μL AllTaq PCR Buffer (QIAGEN; Venlo, Netherlands), 0.5 μL AllTaq DNA Polymerase, 0.25 mM/L dNTPs, 20 μg of Ultra BSA, 0.6 μL of a 1:10,000 solution of SYBR Green I dye, 0.4 μmol/L of forward and reverse primer, 2 μL of DNA and certified DNA free water (QIAGEN) made up to 25 μL total volume. Mastermix was dispensed manually, and qPCR was performed on a CFX96 Touch Real‐Time PCR Detection System (Bio‐Rad, California, USA) using the following conditions: initial denaturation at 95°C for 5 min, followed by 40 cycles of 30 s at 95°C, 30 s at 54°C, and 45 s at 72°C, with a final extension for 10 min at 72°C. All duplicate qPCR products from the same subsample were combined prior to library pooling. The sequencing library was made by mini‐pooling amplicons based on similar qPCR Cq values, which were then quantified (Agilent TapeStation) and combined into equimolar ratios and sequenced on an Illumina Miseq platform (Illumina; San Diego, USA). The libraries were size selected (160–450 bp) using a Pippin Prep (Sage Science, Beverly, USA) and purified using the Qiaquick PCR Purification Kit (Qiagen; Venlo, Netherlands). The volume of purified library added to the sequencing run was determined by quantifying the concentration (Murray, Coghlan, and Bunce 2015) using a Qubit 4 fluorometer (ThermoFisher Scientific). The library was unidirectionally sequenced (single‐end reads) using a 300 cycle MiSeq V2 Reagent Kit and standard flow cell.

### 2.8 Molecular Controls

PCR plates included blank laboratory extraction controls (extraction reagents used with no DNA template), PCR negative controls (2 μL of deionized water used rather than DNA template), and positive controls (West Australian Dhufish; *Glaucosoma hebraicum* ; a sub‐tropical/temperate fish species not typically found in the study area). All three positive PCR controls detected our control species with absolute or high confidence (0.99), at high read counts (648,659, 110,333, and 528,723) providing reference from a known tissue sample (see Section 2.9 for details on species identification). The detection of our positive control was only found in five samples and at very low read counts (3, 10, 12, 17, and 22). Likewise, our three negative PCR controls contained very low read counts (1, 3, and 6) of fish species variants, as did our two extraction controls (1 and 1). We determined that a read count of > 24 was required to confirm a positive species detection within a sample because this number of reads was slightly higher than our highest control read of 22.

### 2.9 Taxonomic Assignments

Our DNA sequence data processing was done using the Ampliseq pipeline and directly followed the procedures described at https://nf‐co.re/ampliseq/2.11.0/ (Straub et al. 2020) using all default settings and these methods are briefly outlined here. Data generated by Illumina sequencing were quality controlled using FastQC (provides a simple way to identify problems in the data) and trimmed using Cutadapt (finds and removes adapter sequences, primers, and other unwanted sequences), resulting in the total reads being reduced from 6,547,632 to 6,302,279 with an overall median read length of 205. An initial amplicon sequence variants (ASV) table (999 ASV's) was produced and taxonomically classified using DADA2 (which uses a Bayesian classifier method to assign taxonomy by comparing to a set of assigned sequences from a reference file and also provides a confidence value for the resulting classification; Callahan et al. 2016; https://benjjneb.github.io/dada2/assign.html) based on a reference database built on 8 March 2021 (produced using ecoPCR in OBITools [Boyer et al. 2016] to extract all sequences from the European Molecular Biology Laboratory matching our universal fish primer assay). As our database was built in 2021, all sequence variants were aligned with BLASTn against the GenBank database on February 7, 2024, to check for updated reference sequence deposits and our classification criteria are outlined below. Our sequencing run contained 20 unusable samples due to known human errors in the field (e.g., sponge or filter membrane dropped on the counter or uncertainty whether the OCD had been opened at depth), which accounted for 827,445 reads and 104 variants. Based on our controls, we eliminated all occurrences of taxa found with < 24 reads in a given sample, further reducing our ASV table to 736 variants with a total read count of 4,168,916 (excluding controls). All taxa with no close matches (≤ 80% identity match to a reference sequence) were eliminated from the final analyses, as were taxa not typically found in the sample area (two assigned species: *Bostockia porosa*, a freshwater fish that does spawn in small tributaries in August and September after winter rains, and *Platycephalus caeruleopunctatus*, a southeastern Australian species). Our final ASV table consisted of 633 variants with a total read count of 3,948,250 and an average read count per sample of 37,602. In general, taxa were classified in order if their percent identity to the reference sequence was between 80% and 90%, family for matches ≥ 90% and < 95%, genus for matches ≥ 95% and < 97, and species if ≥ 97%. However, if an equal percentage identity was attributed to multiple species, then the taxa were assigned to the genus. We provide our final ASV table with taxonomic classifications, best match percentage, and sequence in Appendix S3. Most species level assignments had a ≥ 99% best match percentage. We also provide a list of documented depth ranges for all detected taxa as reported in the Codes for Australian Aquatic Biota (www.cmar.csiro.au) and Fishes of Australia (www.fishesofaustralia.net.au), allowing us to evaluate if our eDNA detections are within the expected depth range (Appendix S4). As depth ranges can be specific to species, in the event that a taxon could only be identified to genus or family, we use the depth ranges of species known to the area within the identified genus or family to determine the most likely depth range.

### 2.10 Statistics

The number of species detected from the three sponge pieces was combined for data analysis to compare against the filtered 1 L samples and rinsed sponge samples. We used an analysis of variance for correlated samples on the number of fish taxa detected by the sample processing method, followed by a Tukey Honest Significant Difference to determine if all methods detected an equal number of fish. We used a permutation MANOVA (adonis) to examine differences in the Family of taxa present from different depth range categories at each site by processing method (all treated as fixed factors). We also performed a nonmetric multidimensional scaling analysis (NMDS) to visually represent the similarities in fish families between depth categories by sites (distance—“jaccard” and permutations—10,000, which ignores joint absences and focusses on the proportion of shared families), where both sites (communities by depth) and species (by family) are overlayed on the same NMDS space. We include ellipses in our NMDS plot based on the standard error for each depth category at a confidence level of 0.9999. Statistics and graphics were produced using Microsoft Excel, R Studio (version 4.1.1; R Development Core Team 2021; “adonis” and “metaMDS” from the R package vegan; https://github.com/vegandevs/vegan, version 2.5‐7), Inkscape (https://inkscape.org/), and Venn diagrams were produced online (http://bioinformatics.psb.ugent.be/webtools/Venn/).

## 3 Results

### 3.1 Comparison of Sample Processing Techniques

A total of 3,948,250 reads were taxonomically assigned to at least order or lower. Of these, 1,257,727 were from the active filtration of the 1 L water samples remaining in the OCD sampler, while the remaining 2,690,523 resulted from detections associated with the processing of the carbon sponge (1,013,366 from the rinsed sponge, and 1,677,157 from the sponge pieces) in the OCD sampler. The number of reads by depth category were 2,042,751, 980,432, and 925,067 for continental shelf, mid‐slope, and abyss, respectively. Taxa were assigned to a total of 37 orders across 87 families, resulting in 193 unique assignments, of which the majority (60%) were also seen in the trawl catches conducted over the entire Gascoyne survey (Table 1).

**TABLE 1:** Fish species detected at depth on the carbon sponge pieces (S), rinsed sponge (R), and by active filtration of 1 L of water on a cellulose ester membrane (A) obtained from the integrated deep tow eDNA sampler.

Order	Family	Species	Method	Depth	Reads by depth	Recorded in catch
Acanthuriformes	Acanthuridae	*Acanthurus* sp.	R	C	3619	No
		*Naso mcdadei*	S	C	540	No
Acropomatiformes	Acropomatidae	*Acropoma* sp.	A,R	M	4583	*leobergi*
		*Doederleinia berycoides*	A,S	M,A	294, 36	Yes
		*Synagrops japonicus*	A	A	2435	Yes
		*Synagrops* sp.	A	C	34	Yes
	Banjosidae	*Banjos banjos*	A,R	C,A	437, 24,757	Yes
	Malakichthyidae	*Malakichthys griseus*	A,S	C,M,A	223, 54,593, 17,184	No
	Pempheridae	*Parapriacanthus ransonneti*	A	C	827	No
		Unidentified Acropomatiformes	S	C,M	16,930, 91	
Alepocephaliformes	Alepocephalidae	*Bathytroctes* sp.	A	A	286	*breviceps* & *squamosus*
		*Herwigia kreffti*	A	A	40	Yes
		*Narcetes* sp.	S	M	161	*erimelas* and *lloydi* and *stomias*
		Unidentified Alepocephalidae	A	M	7295	
Anguilliformes	Congridae	*Bathyuroconger* sp.	R	M	355	*Parvibranchialis* and cf. *vicinus*
		*Conger* sp.	A	M	316	No
		Unidentified *Gnathophis*	A,R	C,A	11,834, 20,668	*Melanocoelus* and two unidentified species
	Muraenidae	*Gymnothorax mucifer*	S	C	3647	Yes
		*Gymnothorax pseudothyrsoideus*	S	C	491	No
	Serrivomeridae	Unidentified *Serrivomer*	A	C,A	311, 3104	Yes
	Synaphobranchidae	*Synaphobranchus affinis*	A,R,S	C,M,A	1992, 19,625, 1671	Yes
		Unidentified Synaphobranchidae	A,R	M,A	196, 3087	
Aulopiformes	Bathysauridae	*Bathysaurus ferox*	A,R	M	2082	Yes
	Chlorophthalmidae	Unidentified *Chlorophthalmus*	A,R,S	C,M,A	105, 89, 8004	*nigripinnis*
		Unidentified Chlorophthalmidae	A	A	346	
	Ipnopidae	Unidentified Ipnopidae	R	C	3331	
	Paralepididae	Unidentified Paralepididae	A,S	C,M,A	128, 65, 2163	
	Synodontidae	*Saurida wanieso*	A,R	M	2385	cf. *filamentosa* and *grandisquamis* and *undosquamis*
		*Trachinocephalus trachinus*	A	C	104	No
		Unidentified Synodontidae	A,R,S	C,A	332,356, 6857	
		Unidentified Aulopiformes	S	C	31	
Beloniformes	Exocoetidae	*Parexocoetus brachypterus*	R	C	1071	No
Beryciformes	Berycidae	*Beryx splendens*	A,R	M	6576	No
Carangiformes	Carangidae	*Alectis ciliaris*	R	C	673	Yes
		*Decapterus* sp.	A,R,S	C	1655	
		*Decapterus tabl*	A,S	M	206	Yes
		*Selar crumenophthalmus*	R	C	1668	No
	Coryphaenidae	*Coryphaena hippurus*	A	A	946	No
Centrarchiformes	Cheilodactylidae	*Cheilodactylus* sp.	A,R	C	611	No
	Cirrhitichyhyidae	*Cirrhitichthys* sp.	A	C	201	cf. *aureus*
Chaetodontiformes	Chaetodontidae	*Chaetodon assarius*	A	C	1331	Yes
	Leiognathidae	*Equulites* sp.	A,R,S	C	21,317	*elongatus*
		*Unidentified Leiognathidae*	A,S	C	1962	
Clupeiformes	Clupeidae	*Amblygaster* sp.	R	C	519	No
		*Sardinella* sp.	A	C	202	No
		*Sardinops sagax*	A,R,S	C,M,A	29,151, 1716, 48,719	No
	Dussumieriidae	*Etrumeus* sp.	A,R,S	C	61,166	No
	Engraulidae	*Engraulis australis*	A,R,S	C,A	654,230, 7802	No
		*Engraulis* sp.	A,S	C	18,081	No
Ephippiformes	Ephippidae	*Platax orbicularis*	A	C	325	No
Gadiformes	Bathygadidae	*Gadomus pepperi*	A	A	202	Yes
		*Gadomus* sp.	A	A	17,294	cf. *colletti*
	Bregmacerotidae	*Bregmaceros atlanticus*	A	C	26	
		*Bregmaceros* sp.	A,R,S	M,A	37,201, 545	Yes
		Unidentified Gadiformes	A,R,S	C	48,861	
	Macrouridae	*Coelorinchus* sp.	A	C	395	Yes
		*Coryphaenoides* sp.	R	C	196	Yes
		*Malacocephalus laevis*	S	M	249	Yes
		*Nezumia* sp.	A	C	244	Yes
		Unidentified *Trachonurus*	A	A	944	Yes
		Unidentified *Ventrifossa*	A,R,S	C,M,A	15,906, 18,341, 39,517	Yes
		Unidentified Macrouridae	A,R	M,A	2633, 905	
	Moridae	*Antimora rostrata*	A	A	1653	Yes
		*Gadella jordani*	A	M	4955	*macrura*
		Unidentified Moridae	R	M	4825	
Gobiiformes	Gobiidae	*Ptereleotris uroditaenia*	R	C	2647	No
		Unidentified Gobiidae	R,S	C	1695	No
		Unidentified Gobiiformes	A,R,S	C	269,041	No
Holocentriformes	Holocentridae	*Ostichthys japonicus*	A	M	4473	Yes
Kurtiformes	Apogonidae	*Siphamia* sp.	A,S	C	4928	*tubifer*
		Unidentified Apogonidae	A,R,S	C	19,648	
		Unidentified Kurtiformes	A,R	C	504	
Labriformes	Labridae	*Bodianus* sp.	R,S	C	7694	*Izuensis* and *solatus*
		*Choerodon cauteroma*	A	C	1635	Yes
		*Choerodon gymnogenys*	A	C	469	*albofasciatus*
		*Coris caudimacula*	A	C	41	No
		*Thalassoma* sp.	A	C	749	No
		Unidentified Labridae	A,R	C	4130	
		Unidentified Labriformes	A,R	C	190,261	
Lophiiformes	Antennariidae	*Antennarius pictus*	S	M	17,262	Yes
		*Antennarius striatus*	R	C	1604	Yes
	Centrophrynidae	*Centrophryne spinulosa*	S	C	1446	Yes
	Chaunacidae	*Chaunax* sp.	A,R	C,M,A	3209, 85, 20,977	*apus* and *breviradius* and *nebulosus* and *penicillatus*
	Lophiidae	*Lophiomus setigerus*	A	C,A	739, 5772	Yes
		Unidentified Lophiiformes	A	A	9199	
Lutjaniformes	Haemulidae	*Diagramma* sp.	A,R,S	C	1831	*pictum*
	Lutjanidae	*Aprion virescens*	A	C	677	No
		*Etelis* sp.	A	M	4601	*boweni*
		*Pristipomoides multidens*	A,R,S	C,M,A	4800, 30,620, 321	Yes
		*Pterocaesio digramma*	A,R,S	C	2638	No
Myctophiformes	Myctophidae	*Diaphus garmani*	A,R,S	M	31,604	Not yet confirmed
		*Diaphus lucidus*	R	M	799	Not yet confirmed
		*Diaphus watasei*	A,R,S	C,M	2384, 7160	Yes
		*Hygophum hygomii*	R	A	5228	*proximum*
		*Lampadena luminosa*	A	M	1034	Yes
		*Lobianchia gemellarii*	A	A	8631	No
		*Triphoturus nigrescens*	A	M	233	No
		Unidentified *Diaphus*	A,R	M,A	11,753, 71,226	
		Unidentified Mytophidae	A,R,S	C,M,A	180, 304,095, 22,695	
	Neoscopelidae	*Neoscopelus macrolepidotus*	A,R,S	M,A	17,872, 12,251	Yes
		*Neoscopelus* sp.	A	A	3214	*microchir*
Notacanthiformes	Halosauridae	*Aldrovandia affinis*	A,R,S	C,M,A	63, 1272, 21,291	Yes
Ophidiiformes	Ophidiidae	*Acanthonus armatus*	A	M	601	Yes
		Unidentified Ophidiidae	A	A	11,634	
Osmeriformes		Unidentified Argentiniformes	A	M,A	301, 1974	
Perciformes	Lethrinidae	*Gymnocranius grandoculis*	A	C	228	Yes
		*Gymnocranius microdon*	A	C	272	No
	Mullidae	*Parupeneus chrysopleuron*	A,R,S	C,M,A	54,759, 45,878, 38,631	Yes
		*Parupeneus* sp.	A,R,S	C,M	15,990, 2364	*heptacanthus*
		*Upeneus* sp.	A,R	C,M,A	18,736, 114, 17,203	*subvittatus*
		Unidentified Mullidae	A,S	C	12,256	
	Opistognathidae	*Opistognathus* sp.	A	C	470	No
		Unidentified Opistognathidae	A	C	337	
	Pinguipedidae	*Parapercis haackei*	A	C	198	No
	Platycephalidae	*Onigocia sibogae*	A	C	58	No
		*Onigocia spinosa*	R,S	C	9487	Yes
	Pomacentridae	*Chromis* sp.	A,R	C	6027	*mirationis* and *sahulensis* and *westaustralis*
		*Pristotis obtusirostris*	A,R,S	C	31,600	No
	Pseudochromidae	*Pseudochromis* sp.	A	C	33	No
	Scorpaenidae	Unidentified Scorpaenidae	A,S	C,M	401, 8249	
	Serranidae	*Epinephelus coioides*	A	C	56	No
		*Epinephelus fasciatus*	A	C	903	No
		*Epinephelus rankini*	A,S	C,M	3223, 70	No
		*Epinephelus rivulatus*	A	C	311	No
		*Luzonichthys waitei*	A,R	C	3439	Not yet confirmed
		*Plectranthias japonicus*	A	M	430	Yes
		Unidentified Serranidae	A	C	567	
	Sillaginidae	*Sillago ingenuua*	A	C	6802	No
		*Sillago robusta*	A,S	C	2347	No
	Triglidae	Unidentified *Lepidotrigla*	A,R,S	C,M,A	17,201, 57,420, 5025	cf. *japonica* and *spiloptera* and unknown
		Unidentified Perciformes	A,R,S	C,M,A	9513, 668, 8260	
Pleuronectiformes	Paralichthyidae	Unidentified Paralichthyidae	A,R,S	C,A	7480, 31,930	
	Poecilopsettidae	*Poecilopsetta* sp.	A	C,M	75, 237	*colorata* and *plinthus* and unknown
Priacanthiformes	Priacanthidae	*Priacanthus sagittarius*	A	C,M	119, 12,865	Yes
		*Priacanthus* sp.	A,S	C,M	120, 6401	*hamrur*
Scombriformes	Gempylidae	*Promethichthys prometheus*	A	C	381	Yes
		*Thyrsitoides marleyi*	S	A	13,147	Yes
	Nomeidae	*Cubiceps whiteleggii*	S	C	42	Yes
	Scombridae	*Auxis thazard*	A,R,S	C,M	3751, 1046	No
		*Euthynnus affinis*	A,S	C	2327	No
		*Katsuwonus pelamis*	A	A	31,340	No
		*Scomber* sp.	A,R,S	C	11,692	No
	Scombrolabracidae	*Scombrolabrax heterolepis*	A	M	229	Yes
	Trichiuridae	*Aphanopus* sp.	S	C	893	Yes
		*Trichiurus lepturus*	A,R,S	C,M	13,801, 7505	cf. *lepturus*
		Unidentified Trichiuridae	A	C,M	308, 585	
		Unidentified Scombriformes	A	C	142	
Scorpaeniformes	Dactylopteridae	Unidentified Dactylopteridae	R	A	2797	
	Neosebastidae	*Neosebastes* sp.	R	A	6326	*occidentalis*
	Scorpaeniidae	*Ectreposebastes* sp.	A	A	6295	*niger*
		Unidentified Scorpaeniidae	A,S	A	10,947	
Spariformes	Lethrinidae	*Gymnocranius grandoculis*	A,R	C,A	81, 520	Yes
		*Lethrinus nebulosus*	A,R	C,M	1234, 25,853	No
		*Lethrinus obsoletus*	A	C	73	No
	Sparidae	*Argyrops* sp.	A	C	884	*bleekeri*
		*Dentex carpenteri*	A,R,S	C,M,A	773, 50,142, 14,525	Yes
Stomiiformes	Gonostomatidae	*Cyclothone obscura*	S	A	4936	Not yet confirmed
		*Cyclothone pallida*	A	A	4369	Not yet confirmed
		*Cyclothone pseudopallida*	A	A	19,656	Not yet confirmed
		*Cyclothone* sp.1	A	M	1096	Not yet confirmed
		*Cyclothone* sp.2	R	A	848	Not yet confirmed
		*Gonostoma atlanticum*	A	M,A	12,509, 9994	Yes
	Sternoptychidae	*Argyropelecus affinis*	A	A	9950	No
		*Sternoptyx diaphana*	A,R,S	M,A	96, 14,188	Yes
		Unidentified Sternoptychidae	A,R	M	1227	
	Stomiidae	*Astronesthes chrysophekadion*	A	M	1110	No
		*Idiacanthus* sp.	R	M	44	*fasciola*
		Unidentified Stomiidae	A,R	M,A	1868, 22,141	
		Unidentified Stomiiformes	A,S	M,A	22,341, 14,022	
Tetraodontiformes	Balistidae	*Abalistes stellatus*	A,S	C,M	79, 1644	No
		*Sufflamen fraenatum*	A	C	44	No
	Ostraciidae	*Tetrosomus concatenatus*	A,S	C,M	194, 4725	No
	Tetraodontidae	*Arothron hispidus*	A	C	68	No
		*Canthigaster rivulata*	A,R	C	13,914	Yes
		*Lagocephalus sceleratus*	A	C	1100	*Lagocephalus* sp.
		*Lagocephalus suezensis*	A	C	3781	Yes
		*Torquigener brevipinnis*	A	A	32,596	No
Trachichthyiformes	Diretmidae	*Diretmoides* sp.	A,R,S	C,M,A	1543, 91,051, 77,457	*vereginae*
		*Diretmus* sp.	A,S	M	250	No
	Trachichthyidae	*Gephyroberyx darwinii*	A	M	581	Yes
		*Hoplostethus mediterraneus*	A	A	4629	cf. *shubnikovi* and unknown
		*Hoplostethus* sp.	A	M	1482	
		Unidentified Trachichthyiformes	R	M	62	
Zeiformes	Oreosomatidae	*Allocyttus* sp.	A	A	2906	*verrucosus*
	Parazenidae	*Parazen pacificus*	R	C	117	Yes
	Zeidae	*Zenopsis nebulosa*	R	A	1261	Yes
	Zeniontidae	*Zenion* sp.	A	C	166	Yes; unknown
Carcharhiniformes	Carcharhinidae	Unidentified Carcharhinidae	S	C	20,992	
	Triakidae	*Mustelus* sp.	A	A	6103	*ravidus* and *stevensi*
Myliobatiformes	Plesiobatidae	*Plesiobatis daviesi*	A	A	122	Yes
Rajiformes	Arhynchobatidae	Unidentified Arhynchobatidae	A,R,S	C,M,A	1339, 2604, 42,029	*Insentiraja subtilispinosa* and *Notoraji hirticauda* and *Pavoraja alleni*
	Rajidae	*Dipturus* sp.	A,R,S	C,M,A	280, 2924, 12,632	*healdi* and cf. *wengi*
Squaliformes	Etmopteridae	*Etmopterus bigelowi*	R	M	12,165	Yes

*Note:* The depth is categorized as continental shelf (C; 0—200 m), shelf break to mid‐slope (M; 200–1000 m), and mid‐slope to abyss (A; 1000–4000 m). Taxa are listed in alphabetical order first by class, order, family, and species (Class Actinopterygii precedes Class Chondrichthyes). Taxa recorded in the catch data are indicated and where possible, we include species names that were identified from the catch data that were not resolved by eDNA.

We had 21 sites where we could compare the taxa detected by each of the three processing methods (two sites had at least one method that produced no results) and found no significant difference between the combined sponge pieces and the rinsed sponge, although both these methods detected significantly less taxa than filtering the 1 L water sample remaining in the OCD sampler (one‐way analysis of variance for correlated samples, *F* = 8.17, df = 2, *p* = 0.001; Tukey HSD Test, HSD [0.5] = 5.18, Filtered 1 L vs. Sponge Pieces *p* < 0.01, Filtered 1 L vs. Rinsed Sponge *p* < 0.01, and Sponge Pieces vs. Rinsed Sponge *p >* 0.05; Figure 4 shows an average number of taxa detected by processing method ± standard deviation). Of the 193 unique taxa detected, 154 were from filtered 1 L samples, 81 were from rinsed sponge samples, and 70 were from sponge pieces. There were only 35 taxa that overlapped with all processing methods, and each method detected taxa not present in the others (Figure 4). No fish were detected in four of the filtered 1 L samples, six of the sponge pieces, and four of the rinsed sponges, which were all abyss sites except one mid‐slope site. The absence of fish detection may indicate there was little field contamination.

**FIGURE 5:**
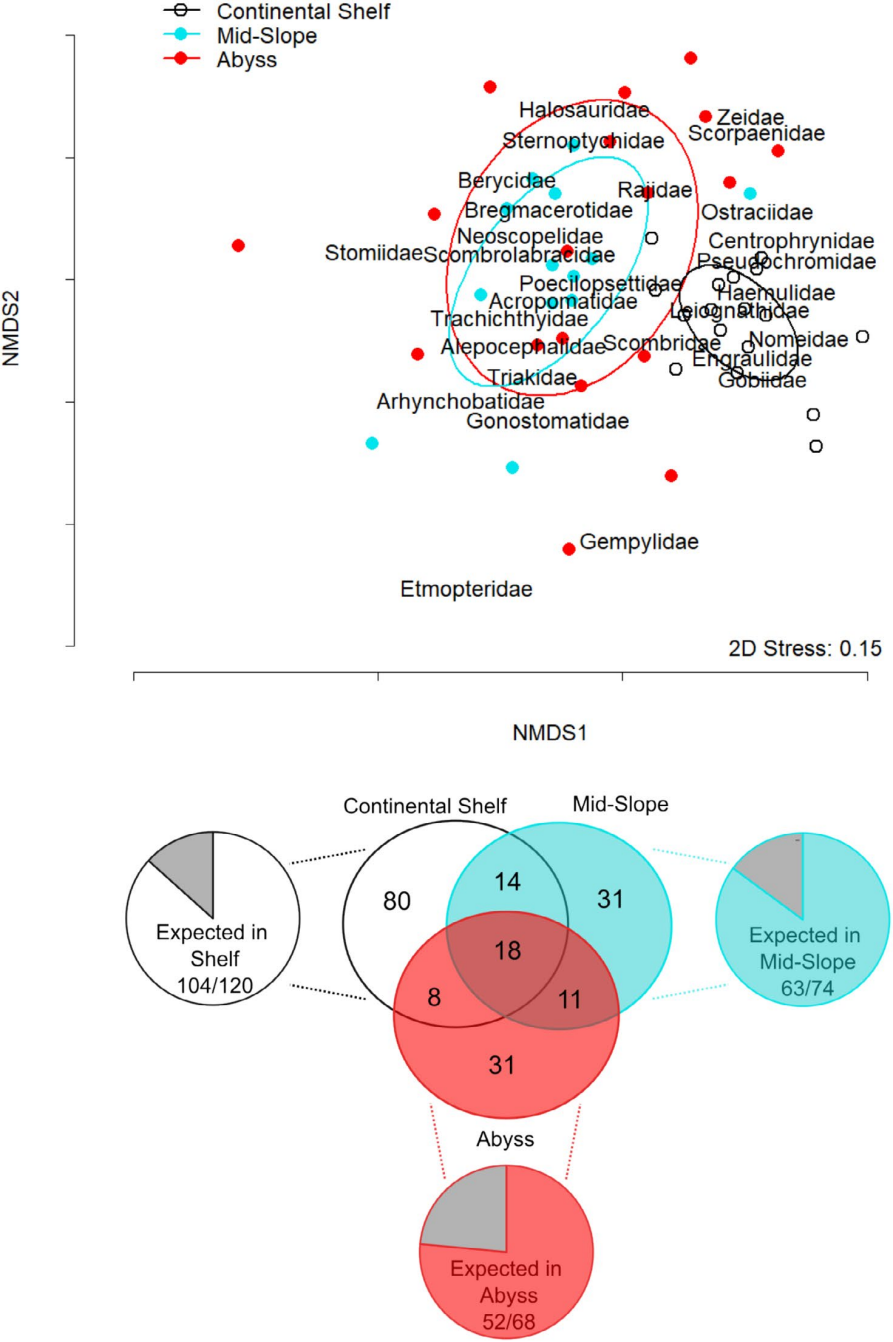
Nonmetric multidimensional scaling plot (NMDS) of the presence/absence of fish families (distance = jaccard, which ignores joint absences and focusses on the proportion of share families) by depth category (black line with open white circle = continental shelf, blue line with solid blue circle = mid‐slope, and red line with solid red circle = abyss). Lines are ellipses based on the standard error for each depth category at a confidence level of 0.9999. Venn diagram depicts the number of taxa detected in each depth category and pie charts show the proportion (in color) of the taxa expected to be found in that depth category, with the gray shaded area showing the proportion of taxa not expected. Data for this figure comes from the 17 sites where all sample processing methods detected fish.

We had 17 sites where all three sample processing methods detected fish and could be used in our permutational multivariate ANOVA to compare the effects of depth category, sample processing method, and site on the presence of fish by family. Both depth category and site were significant factors in the family of fish present (Table 2), with fish families overlapping in mid‐slope and abyss samples, and those found in continental shelf waters showing less overlap (Figure 5; top). Fish families, such as Gobiidae, Engraulidae, Leiognathidae, Pseudochromidae, and Haemulidae, were detected in continental shelf samples, all of which are typically found on the continental shelf or mostly inhabit shallow waters (Appendix S4). In contrast, fish from families, such as Halosauridae, Sternoptychiadae, Stomiidae, Neoscopelidae, and Gonostomatidae, are typically only found in the mid‐slope or abyssal waters (mesopelagic/bathypelagic depths), which was reflected in the eDNA results. Most taxa were detected within their expected depth range (Figure 5; bottom): 87% (104/120) in continental shelf waters, 85% in mid‐slope waters, and 76% in abyss waters (52/68). Nevertheless, there were a few taxa from the family Macrouridae and Myctophidae that were detected in continental shelf waters that are typically deeper water species or mesopelagic, although most unexpected detections were of shallow water species found in deep waters (see Appendix S4 for a detailed list of all taxa by documented depth range).

**TABLE 2:** Permutational multivariate ANOVA results (permutations = 999).

Factor	Df	SumsOfSqs	MeanSqs	*F* model	*R* ^2^	*p*
Depth category	2	3.407	1.704	4.945	0.175	0.001*
Sample processing method	2	0.667	0.333	0.968	0.034	0.532
Site	1	0.610	0.610	1.771	0.031	0.021*
Depth category × sample processing method	4	0.951	0.238	0.690	0.049	0.980
Residuals	40	13.783	0.345		0.710	
Total	49	19.418			1.000	

*Note:* An * indicates significance.

## 4 Discussion

This study introduces the successful design and manufacturing of a passive eDNA sampler, the OCD, that can be attached to a preexisting deep‐sea towed underwater camera system. The study also compared three different methods for processing the captured eDNA from the sampler, and their combined ability to corroborate species identifications obtained from subsequent trawl catches in the study area, providing valuable insight for improvements to optimize DNA collection. Finally, we provide a biological interpretation of the resulting data demonstrating the usefulness of such technology to understanding ocean environments, using fish species as our example organisms. Here we discuss design and manufacturing lessons, ideas for increased eDNA capture efficiency, how to establish appropriate field controls, and how this technology could advance our scientific understanding in ocean studies.

### 4.1 Design and Fabrication Lessons

The OCD was deployed to depths of almost 4000 m, where the resulting pressures are approximately 40,152 kPa, exerting immense pressure on the actuator, lid, and ends. A prior commissioning voyage observed air ingress in the sampling chamber causing implosion and complete unit failure, necessitating the pressure relief valve in the lid. We found that the elastic restraining bands used to secure the lid in position stretched over consecutive deployments preventing the lid from sealing completely. This was fixed in the field by using two stainless steel hose clamps around both ends of the box to secure the lid. The rubber ends that abutted the O‐ring seals also deteriorated after repetitive bleach and pressure cycles causing leakage, which was also fixed in the field by replacing them with commercially available soft plastic pads. The material choice of the sampler, high‐density polyethylene, proved not durable enough for the continual installation and removal from the camera system, sustaining multiple breakages and requiring a rebuild of the sampler and lid in anodized aluminum. The Proflow quick‐release mounts, though convenient, would often not latch and failed after multiple deployments requiring replacement with stainless steel fittings. Finally, we realized that the actuator arm was not creating equal closing pressure across the doors and needed continual adjustment to seal. This will be addressed in an upgraded version of the OCD using articulated door hinges to spread the closing force.

### 4.2 DNA Collection Optimization

Filtering the 1 L water sample remaining in the OCD detected more taxa on average than either the sponge pieces or rinsing the sponge, with only 35 taxa overlapping for all methods, demonstrating that the sample processing method can have a substantial effect on taxa detections. Since all methods had many unique taxa, especially filtering of the OCD water, our results imply that increased eDNA capture efficiency should be achieved by combining all processing methods or, alternatively, finding a method that more effectively extracts eDNA from the water and collection material simultaneously. We hypothesized that eDNA would be concentrated on the sponge, but our results clearly show that eDNA in the water and on the sponge can be different. Theoretically, the idea of towing the open sampler over the length of the transect would result in increased eDNA becoming enmeshed in the sponge. This is an area for improvement in our OCD sampler and offers a challenge to determine what materials would be best suited to use inside the sampler to increase capture efficiency. Many alternative materials (i.e., cotton, nylon, hemp, cellulose, electrospun nanofibers, chitosan, clay, and sand) have been successfully used in passive eDNA capture (Bessey et al. 2022; Kirtane, Atkinson, and Sassoubre 2020) and will need to be trialed to improve the OCD. The flexible design of the box makes this possible because any number of filter holders and membranes can be manufactured to fit the internal chamber of the OCD. For example, the design of a mini‐bongo net insert for the OCD sampler may be a possible solution for increasing the eDNA capture efficiency, and experiments trialing such methods are currently underway.

Another possible reason for the limited ability of the sponge to capture eDNA could be that the orientation, thinness, and surface area exposure were not optimal in relation to the water flow through in the sampler. It is reasonable to think the water would take the path of least resistance through the sampler, thereby providing limited ability for eDNA particles to adhere to the sponge. A possible solution could be to use a series of deflectors to direct the flow of water toward the collection material to increase contact. Different deflector angles and porosity (e.g., perforated or solid) have been used to increase turbine performance, redirect and sequester oil from oil spills, and mitigate river scouring indicating they are useful in many applications (Obead and Sahib 2023; Prasetyo et al. 2018; Eryuzlu and Hausser 1977). Alternatively, it is possible that the sponge did collect eDNA but that the eDNA was released into the OCD water when removing the sponge from the device resulting in the 1 L samples containing the most taxa. Future studies could test this by deploying the OCD with and without the sponge insert in the same location, or by comparing the 1 L OCD samples with 1 L Niskin samples obtained from the same location. Nevertheless, any eDNA contained in the water within the OCD when it closes did come from the target depth along the transect and therefore remains a valid part of the sample.

### 4.3 Establishment of Field Controls

Fish eDNA was not detected at one abyssal site and one mid‐slope site, neither from the OCD water nor sponge samples. This indicated that either the OCD did not open due to malfunction or ambient eDNA concentrations were below detection limits. The latter is a more plausible explanation, as there was no indication of an OCD failure, and an imagery specialist noted that no fish were seen on the deep tow camera footage of the abyssal site. The lack of fish eDNA detection also indicates there was no ingress of water through the valve or O‐rings. Although unplanned, this observation suggests that the OCD does not suffer from unrecognized contamination issues—an important aspect of field control that needs to be incorporated into future studies and use of the OCD sampler. A suitable field control would be to deploy the OCD sampler and ensure it remains closed, which will provide an opportunity to test the deionized water and sponge capture material in the sampler for zero species detections after it has endured the entire deployment. Conducting such field controls would be best done in each different environment that is being sampled, particularly over a gradient of increasing depth, where both species composition and the risk of leakage from increasing pressure will change. To determine contamination issues arising through OCD sampler loading and preparation, as opposed to deployment procedures, future testing should also examine the deionized water used on the boat, the sponge capture material prior to use, and testing of the bleach water that was used to sterilize the sampler and other associated filtering apparatus. Indeed, these smaller, yet critical details in establishing effective negative controls will assist in quality assurance and more robust eDNA sampling procedures (Sepulveda, Birch, et al. 2020).

### 4.4 Advancement of Scientific Understanding

Although our study was designed as a proof of concept for the OCD, it also provided valuable scientific insight about the study area. We were able to detect 193 taxa of fish species from OCD samples and corroborate 60% of these identifications with trawl catch data conducted concurrently in the study area. Like all samplers, trawl gear is selective, and therefore we anticipated catch data and eDNA data would provide complementary rather than substitutive pictures of fish diversity, but that a comparison of data would still provide some level of confidence for detection plausibility. We augmented and validated the comparisons by including information on the depth range of detected and known species inhabiting the area (Appendix S4) and found that, in all depth categories, our detections were plausible most of the time (> 75%) and were able to partition fish assemblages by depth category. Depth‐related patterns of environmental DNA have been observed in previous studies of deep‐sea fishes and could contribute to biodiversity surveys in the deep ocean (McClenaghan et al. 2020). Nevertheless, more insight into why some species were found outside of their typical depth zone is needed before the potential of the OCD can be fully assessed. A possible explanation for some species, particularly mesopelagic groups, such as Myctophidae detected at the abyssal seafloor, could be the result, first, of eDNA settling in the deep ocean where, second, it is persistent for long periods because cold temperatures make DNA less susceptible to degradation (McCartin et al. 2022). If the abyssal near seabed water layer is a sink for DNA particles from epipelagic, mesopelagic, and bathypelagic fauna, then eDNA sampling may be powerful for providing an integrated vertical or regional picture of total biodiversity rather than a snapshot of local diversity. This possibility is strongly suggested by our data. Net samplers and cameras provide different derived ecological metrics for deep‐sea diversity that may be complementary but not interchangeable, and the choice of sampler(s) needs to be determined by survey objectives and possible trade‐offs (Williams, Althaus, and Schlacher 2015). The utility of eDNA methods will be similarly dependent on the nature and spatial scale of the question being asked, e.g., in the deep sea, they may be well‐suited to surveys of biodiversity but poorly suited to studies of vertical structure and diel migration. Importantly, eDNA methods have a low environmental impact and are therefore arguably more ethical than extractive methods, particularly in areas of high conservation importance or heightened sensitivity, and are cost‐effective, e.g., the OCD piggybacks on another sampling tool and so adds no additional cost to the operational deployment time of a field program. It is also likely that the OCD will detect more species than non‐extractive image‐based techniques because it operates at a lower taxonomic resolution. Future paired experiments using the OCD and both extractive and non‐extractive methods should also include image analysis specialists, further advancing our scientific understanding of how non‐extractive video techniques pair with eDNA and to what level of classification is reasonable and obtainable from image analyses. It is through collaborative efforts and understanding of all methods that we will best equip scientists with the most appropriate techniques to address unanswered questions and help conserve our invaluable ocean environments.

## Author Contributions

**Cindy Bessey:** conceptualization (equal), data curation (equal), formal analysis (equal), methodology (equal), validation (equal), visualization (equal), writing – original draft (equal), writing – review and editing (equal). **Andrew Martini:** conceptualization (equal), funding acquisition (equal), methodology (equal), writing – review and editing (equal). **Alasdair Currie:** conceptualization (equal), methodology (equal), writing – review and editing (equal). **Will Ponsonby:** conceptualization (equal), methodology (equal), writing – review and editing (equal). **Aaron Tyndall:** conceptualization (equal), methodology (equal), writing – review and editing (equal). **Ryan Crossing:** data curation (equal), writing – review and editing (equal). **Vinícius Werneck Salazar:** formal analysis (equal), writing – review and editing (equal). **Kathryn L. Dawkins:** formal analysis (equal), writing – review and editing (equal). **John J. Pogonoski:** conceptualization (equal), data curation (equal), writing – review and editing (equal). **Glenn Moore:** data curation (equal), writing – review and editing (equal). **Nick Mortimer:** methodology (equal), writing – review and editing (equal). **John K. Keesing:** conceptualization (equal), data curation (equal), funding acquisition (equal), methodology (equal), project administration (equal), supervision (equal), writing – review and editing (equal).

## Ethics Statement

The work was carried out with approval under CSIRO Wildlife, Livestock and Laboratory Animal Ethics Committee application number 2021‐25, Australian Fisheries Management Authority permit number 1005478, Department of Primary Industries and Regional Development exemption number 3688, Environment Protection and Biodiversity Conservation Regulations 2000 permit number AU‐COM2022‐565, and Australian Marie Park Activity Permit number PA2021‐00092‐1 and PA2021‐00092‐4.

## Conflicts of Interest

The authors declare no conflicts of interest.

## Supporting information


**Appendix S1.** OCD site deployment details.
**Appendix S2.** OCD standard operating procedures.
**Appendix S3.** Final amplicon sequence variants (ASV) table with taxonomic classifications, best match percentage, and sequence details.
**Appendix S4.** Documented depth ranges for all detected taxa as reported in the Codes for Australian Aquatic Biota (CAAB) & by Fishes of Australia. Pelagic species are highlighted in pink and gray shading denotes the relationship between depth range and depth category.

## Data Availability

We include all relevant data in the Appendices S1–S4, including deployment details, standard operating procedures, taxonomic assignments with DNA sequences, documented depth ranges for all detected taxa, and the final analyzed dataset. All associated files to reproduce this work are available on the CSIRO Data Access Portal at https://data.csiro.au/collection/csiro:63803 and contains our raw fastq.gz (single‐end reads) sequencing data and associated sample sheet with primer and index combinations, R‐script for Figure 5 and associated data, a copy of all Supporting Informations (Appendices S1–S4), as well as the reference database and associated code.
