# Supplementary material for: Design and Validation of an Open–Close Device for Integrated Environmental DNA Sampling Detects A Depth Gradient in Indian Ocean Deep‐Sea Fish Assemblages
Source: Ecol Evol. 2025 Jan 28;15(2):e70902. doi: 10.1002/ece3.70902 (PMC11775381; doi:10.1002/ece3.70902)
Supplement: Supplementary file 1 — Appendix S1. OCD site deployment details. Appendix S2. OCD standard operating procedures. Appendix S3. Final amplicon sequence variants (ASV) table with taxonomic classifications, best match percentage, and sequence details. Appendix S4. Documented depth ranges for all detected taxa as reported in the Codes for Australian Aquatic Biota (CAAB) & by Fishes of Australia. Pelagic species are highlighted in pink and gray shading denotes the relationship between depth range and depth category. [file ECE3-15-e70902-s001.zip › ece370902-sup-0002-AppendixII.docx]

Safe Work Instruction (SWI)


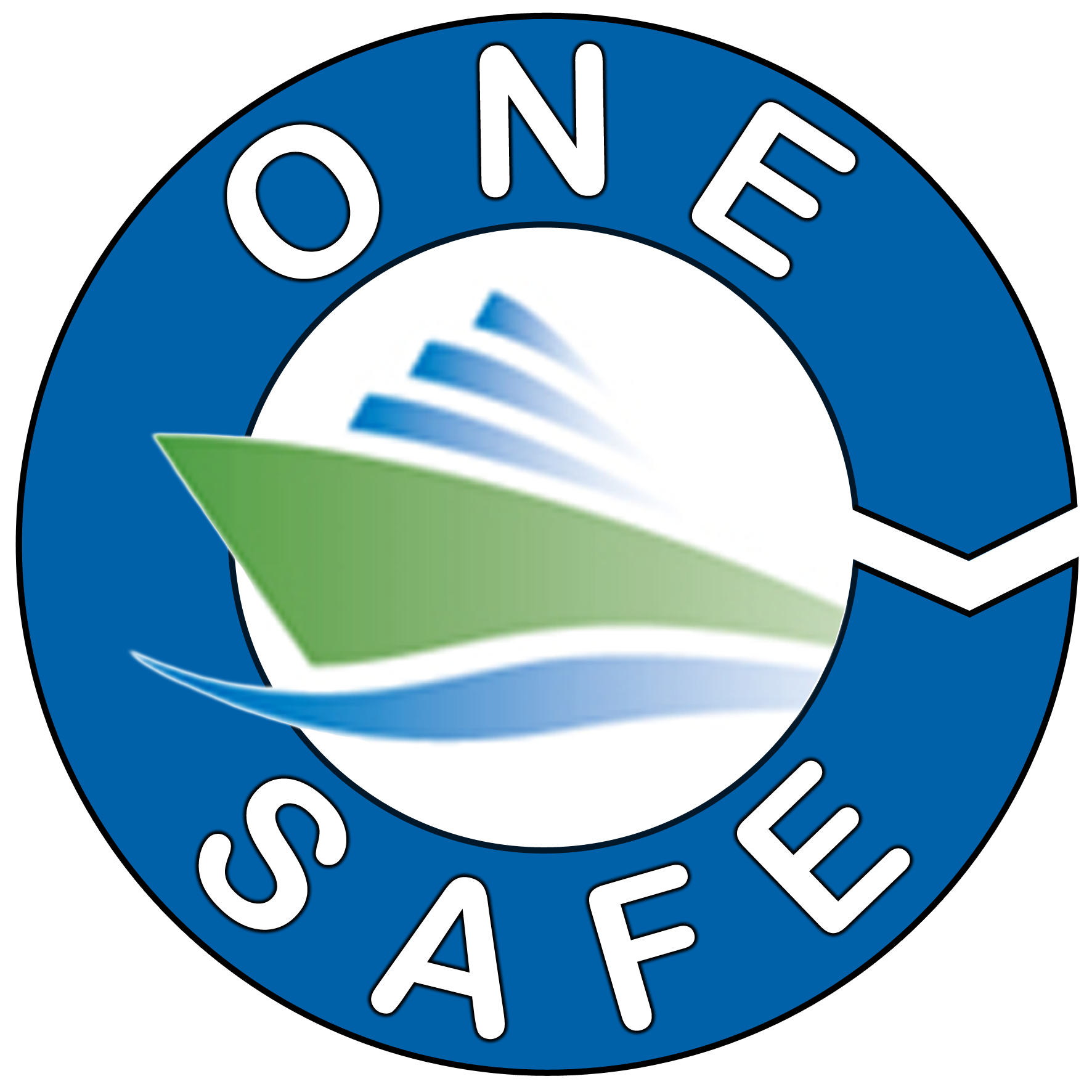


Marine National Facility/RV *Investigator*

RV *Investigator* is a moving platform and exposed to rapidly changing sea conditions. It is a closed community that operates 24/7 with two shifts a day. Hazards that may be a low risk on shore may become high risk on board. SWI describes the safety precautions to ensure all controls are in place to minimise risk, as much practicable. Written by staff with sound, hands-on experience, knowledge of the work and the operational/regulatory requirements. Reviewed and signed by HSE and/or subject matter specialists (RSO for any items licenced with ARPANSA). Approved and signed by the relevant Line Manager. Kept on or adjacent to the plant/equipment or where the work activities are being undertaken.

| **TITLE** | Passive eDNA collection using detachable device mounted on the deep-water tow video |
| --- | --- |
| **What (description)** | This SWI describes the process of taking environmental DNA samples from the detachable passive eDNA collector mounted to the deep-water tow video.  Loading   1. Sterilized (UV), commercially available carbon activated sponge pads (Aqua One ®; 25.4 cm width x 45.7 cm height) are inserted into the open collection device using sterile, gloved hands. 2. Deionized water is then used to completely fill the collection container. 3. The collection device is closed, ensuring no air bubbles remain in the compartment which is evidenced by slight overflow during closure of the collector. 4. The loaded collection device is provided to seagoing instrumentation who are responsible for the instillation and removal of the unit from the camera.   Unloading   1. Open chamber in the dry clean lab using sterile technique with gloved hands. 2. Remove activated carbon sponge from the chamber allowing for excess water to drip back into the collection device. 3. Place activated carbon sponge (sample) in labelled plastic Ziploc bag, removing excess air to conserve space. 4. Place sample in -80°C freezers for storage.   Excess Water Processing   1. Pour water remaining in collection device into a sterile beaker. 2. Filter water over a 0.45µm 47 mm cellulose membrane using a peristaltic pump contained in the eDNA filter kit (see reference documents). 3. Using sterile tweezers, fold cellulose membrane and place in a labelled plastic Ziploc bag. 4. Place sample in -80°C freezers for storage.   Decontamination of Collection Device   1. Place collection device in 10% bleach solution for 20 minutes. 2. Thoroughly rinse collection device in deionized water. 3. Air dry and store closed. |
| **Where (site/location)** | RV Investigator  Dry lab for filter removal and replacement.  Dry lab for filtration of water that was enclosed in the collector  -80°C freezers for storage of sponge filter inserts and filtered membranes. |
| **Who (team/individuals)** | All passive eDNA practitioners using the tow video passive eDNA collector |
| **Business Unit** | NCMI / O&A |
| **Reference documents** | CSIRO eDNA Sampling SOP (attached) |

| **SWI REVIEW (MNF requires a minimum of 12 monthly review)** | |
| --- | --- |
| **Review Frequency** | - This SWI must be reviewed within 12 months of last review date or; - when changes are made to how the activity is undertaken. |

| **BEFORE YOU START** | |
| --- | --- |
| **Competencies and training** | Training required by competent person.  -80˚C freezer induction. |
| **Key hazards** | - Potential for slips, trips, and falls - Due to wet surfaces in CTD room - Contact with extremely cold surface (-80C freezer) - Sea door in CTD room, high sill, and negotiating the door of the clean dry lab. - Pinch Points - When opening and closing the collection device ensure hands and fingers are clear from rim and seals. |
|  | \| **[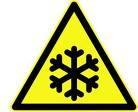](http://www.google.com.au/imgres?imgurl=http://0.tqn.com/d/chemistry/1/0/9/h/lowtemperature.jpg&imgrefurl=http://chemistry.about.com/od/healthsafety/ig/Laboratory-Safety-Signs/Low-Temperature-Warning-Symbol.htm&usg=__W7z3vYn9uUl02lLIYVG4Qv_fSsU=&h=540&w=665&sz=34&hl=en&start=2&zoom=1&itbs=1&tbnid=LUIDZkqLQQckqM:&tbnh=112&tbnw=138&prev=/images?q%3Dcryogenic%2Bwarning%2Bsymbol%26hl%3Den%26sa%3DG%26gbv%3D2%26tbs%3Disch:1&ei=-ndcTY6YC5LRcMGYqLMK)** \| [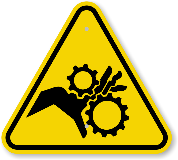](http://www.google.com.au/url?sa=i&rct=j&q=&esrc=s&source=images&cd=&cad=rja&uact=8&ved=0ahUKEwirtuOko_LPAhXMnJQKHWnGCfgQjRwIBw&url=http://www.mysafetysign.com/iso-warning-signs&psig=AFQjCNGV67whS1F5IS52a7Ksbckq9H7tEQ&ust=1477358420472671) \| [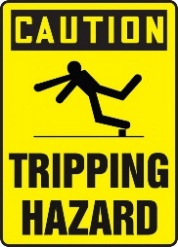](http://www.google.com.au/url?sa=i&rct=j&q=&esrc=s&source=images&cd=&cad=rja&uact=8&ved=0ahUKEwivxKnqpPLPAhWGoJQKHfaYD0oQjRwIBw&url=http://www.accuform.com/safety-sign/caution-caution-tripping-hazard-wgraphic-MSTF667&bvm=bv.136593572,d.dGo&psig=AFQjCNEUeAKUtvwCco4gr358g91JZwa3eg&ust=1477358850883686) \|  \|  \| \| --- \| --- \| --- \| --- \| --- \| \| Low Temperature \| Danger of Pinching \|  \|  \|  \| |
| **Physical controls  (level 2 controls)** | In rough weather, a second person should assist with the dry clean lab and the -80C freezer door (freezer induction instructions). |
| **Personal controls and PPE (level 3 controls)** | \| Lab coat or overalls, enclosed footwear, protective gloves to protect the samples. \| \| --- \| \| \| 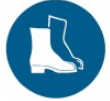 \| 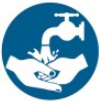 \| 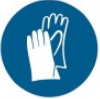 \| \| --- \| --- \| --- \| \| Safety Footwear \| Hand Washing \| Gloves \|  \| 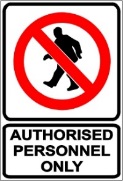 \| 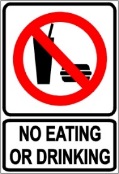 \| \| --- \| --- \| \| |
|  |  |

| **Emergency preparedness and response** | The filtration operations are low risk (1,2).  Emergency response equipment/resources must be in place and you should be familiar with this location and use before you start any activities e.g. first aid kits, fire extinguishers, chemical spill kits, radiation skill kits, cyanide response kits, safety observer, first aid officer.  REPORTING IN THE CASE OF AN EMERGENCY –report to the Vessel Management Team:   - Master via the bridge: #600 - Voyage Manager: #332 - Chief Scientist: #331   IN THE CASE OF EMERGENCY – ACTIVATE SAFETY PLAN (list the actions to be taken in the event of an emergency relevant to the work activities and/or plant and equipment).   - Ensure your own safety - Ensure the safety of those around you - Evacuate the laboratory if noxious odour is identified/ Area is unsafe to remain - Activate signage to close of area - REPORT |
| --- | --- |

| **PROCESS STEPS and SAFETY CONTROLS AT EACH STEP** |
| --- |
| **Cleanliness**  The ship and you are sources of DNA contamination:  Wear gloves and lab coat.  **Process steps**  **Pre-station checks**   1. Make sure -80 ˚C freezer in main deck hallway is turned on! 2. Communicate opening and closing timing of the collection device with seagoing instrumentation operator. This will usually coincide with the start and end point of the tow camera at depth. 3. Label bag containing the sterile filter pad with date, site, depth, and all relevant site information. 4. Set-up the peristaltic pump in accordance with the CSIRO eDNA Sampling SOP (attached).   **Loading the Collection Device**   1. Prepare collection device in the clean dry lab. Be mindful that clothing fibres, hair and skin and anything else landing in the open chamber is potential contamination. 2. Place device in a sterile tray and place on the bench in the clean dry lab. Open chamber using sterile technique with gloved hands and lab coat. 3. Remove sterilized active carbon sponge pad from Ziploc bag and place directly inside the opened collection device chamber. 4. Pour deionized water into the collection device directly over the sponge pad until there is a slight overflow, thereby ensuring the chamber is completely full. This slight overflow of deionized water will be contained in the sterile tray. 5. Close the collection device, ensuring no air bubbles remain in the chamber. A slight overflow should be expected during closure. 6. The loaded, closed, collection device is provided to seagoing instrumentation who are responsible for instillation of the unit onto the tow camera.   **Unloading the Collection Device**   1. Receive collection device and place in tray on the bench in the clean dry lab. 2. Open collection device, then change gloves, ensuring only sterile gloved hands are used when handling the filter sponge. 3. Remove filter sponge from chamber, allowing for excess water to drip back into the collection device. Compress filter pad against itself ensuring all water is released back into the collection device. 4. Place filter sponge (sample) back into original labelled plastic Ziploc bag. Remove excess air from back to conserve space during storage and ensure bag is securely closed. 5. Place labelled sample in -80°C freezer for storage.   **Excess Water Processing**   1. Pour water remaining in collection device into a sterile beaker. 2. Filter water over a 0.45µm 47 mm cellulose membrane using the peristaltic pump contained in the eDNA filter kit (see CSIRO eDNA Sampling SOP; all necessary equipment contained within the eDNA Kit). 3. Using sterile tweezers, fold cellulose membrane and place in a labelled plastic Ziploc bag. 4. Place sample in -80°C freezers for storage.   **Decontamination of Collection Device**   1. Prepare a 10% bleach solution in a yellow collection tube within the sink. 2. Place collection device in the 10% bleach bath for 20 minutes. **To minimize the potential of corrosion, it is imperative that the collection device is never left in the bleach solution for longer than the designated 20 minutes. 3. Thoroughly rinse collection device in deionized water. 4. Air dry the collection device in the clean dry room. 5. Store the collection device in a clean dry space ensuring the lid remains closed.   Keep traffic to a minimum during loading and unloading of filter membranes. |

| **BEFORE YOU FINISH** | |
| --- | --- |
| **Troubleshooting** | All equipment is to be maintained clean by all users. |
| **Waste disposal** | Waste is non-hazardous and can be disposed of by usual ships quarantine protocol. |
| **End of work state** | The collection device is to be bleached, thoroughly rinsed, dried, and stored cleanly with the tow video apparatus. Unless the equipment is required for a subsequent voyage, all eDNA Kits are to be returned to the Ecological Genetics Laboratory in Crawley. |

| **DOCUMENT APPROVAL** | | | |
| --- | --- | --- | --- |
| **Document Owner** Author | name: Cindy Bessey | signature | date |
| **Manager/Supervisor** Approver | name | signature | date |
| **MNF Science Technology Co.**  Reviewer (As required by the activity) | name | signature | Date |
| **MNF Vessel Manager**  Reviewer (As required by the activity) | name | signature | Date |
| **HSE / Specialist** Reviewer (As required by the activity) | name | signature | Date |

**Ensure this SWI is captured on the Prestart Attendance form. Persons signing onto the Prestart Attendance form acknowledge that they understand the instructions listed this SWI and will comply with them.**

| **Version Number** | **Date issued** | **Reviewed by** | **Minor or major change** | **Version Number** | **Date issued** | **Reviewed by** | **Minor or major change** |
| --- | --- | --- | --- | --- | --- | --- | --- |
| 9.0 | 25/9/2019 | Antony Lucas | Major | 10 | 04/05/2021 | Antony Lucas | Major |
| 9.1 | 31/01/2020 | Antony Lucas | Minor |  |  |  |  |
| 9.2 | 31/03/2020 | Antony Lucas | Minor |  |  |  |  |
| 9.3 | 01/12/2020 | Antony Lucas | Minor |  |  |  |  |
